# Classifier-Based Data Transmission Reduction in Wearable Sensor Network for Human Activity Monitoring

**DOI:** 10.3390/s21010085

**Published:** 2020-12-25

**Authors:** Marcin Lewandowski, Bartłomiej Płaczek, Marcin Bernas

**Affiliations:** 1 Institute of Computer Science, University of Silesia, Będzińska 39, 41-200 Sosnowiec, Poland; marcin.lewandowski@us.edu.pl; 2 Department of Computer Science and Automatics, University of Bielsko-Biała, Willowa 2, 43-309 Bielsko-Biała, Poland; mbernas@ath.bielsko.pl

**Keywords:** wireless sensor network, wearable sensors, activity recognition, lifetime, energy consumption, transmission suppression, embedded machine learning

## Abstract

The recent development of wireless wearable sensor networks offers a spectrum of new applications in fields of healthcare, medicine, activity monitoring, sport, safety, human-machine interfacing, and beyond. Successful use of this technology depends on lifetime of the battery-powered sensor nodes. This paper presents a new method for extending the lifetime of the wearable sensor networks by avoiding unnecessary data transmissions. The introduced method is based on embedded classifiers that allow sensor nodes to decide if current sensor readings have to be transmitted to cluster head or not. In order to train the classifiers, a procedure was elaborated, which takes into account the impact of data selection on accuracy of a recognition system. This approach was implemented in a prototype of wearable sensor network for human activity monitoring. Real-world experiments were conducted to evaluate the new method in terms of network lifetime, energy consumption, and accuracy of human activity recognition. Results of the experimental evaluation have confirmed that, the proposed method enables significant prolongation of the network lifetime, while preserving high accuracy of the activity recognition. The experiments have also revealed advantages of the method in comparison with state-of-the-art algorithms for data transmission reduction.

## 1. Introduction

Wireless wearable sensor networks are usually composed of several sensor nodes attached to human body or embedded in clothing [1,2,3]. The sensor nodes can monitor parameters of the body as well as its environment. Communication between the wearable sensor nodes is based on wireless data connections. So far, many applications of the wearable sensor networks have been considered in the literature. These potential applications include healthcare, localization, activity monitoring, sport, fitness, augmented reality, safety, rescue, emergency management, and many others.

Successful use of the wearable sensor networks depends on lifetime of the batterypowered sensor nodes. In order to ensure long network lifetime, the limited energy resources of sensor nodes have to be efficiently utilized. The most energy expensive operation of wireless sensor nodes is data transmission. In contrast, data processing consumes significantly less energy. Reduction of data transmission can lead to considerable energy savings and increased network lifetime [4,5,6]. Thus, effective methods are needed to avoid any unnecessary data transmissions in the wearable sensor networks. The existing data reduction methods are mainly focused on wireless sensor networks that collects sensor readings, execute simple prepossessing tasks, and send the data to a sink or a base station. In contrast, the sensor network considered in this paper is composed of wearable devices that perform more complex tasks related to human activity recognition by using embedded machine learning algorithms.

This paper introduces a method for reducing data transmissions in wearable sensor networks, where a cluster head node collects data from different sensors to recognize human activity in real-time. In this method, the sensor nodes are equipped with binary classifiers that allow them to decide if current sensor readings have to be transmitted to cluster head or not. According to the proposed approach, the data classifiers for sensor nodes are trained with use of machine learning algorithm. An algorithm was elaborated to prepare a training data set, which consists of data readings from sensors and assigned labels. Each label indicates if a given data reading is necessary to correctly recognize the activity of monitored person or not. These labels are determined during tests of a recognition algorithm, which is used by the cluster head node to categorize human activities.

The proposed method was implemented in a prototype of wearable sensor network for activities recognition of persons working in a computer laboratory. The prototype was designed to recognize the human activities, such as sitting, walking, or standing. Moreover, in case of sitting person, it was also recognized where the person is sitting and if the monitor is switched on. During experiments, energy consumption of sensor nodes was measured and lifetime of the wearable sensor network prototype was analysed. Results of the experimental evaluation have enabled detailed comparison of the network lifetime for the proposed approach, and for state-of-the-art transmission reduction methods. Also, the impact of data transmission reduction on accuracy of activity recognition was analysed for the compared methods in real-world experiments.

The paper is organized as follows. Section 2 reviews related works and discusses contribution of this paper. Section 3 presents the proposed approach to reducing data transmission in wearable sensor networks. Experiments and their results are described in Section 4. Finally, conclusions are given in Section 5.

## 2. Related Works and Contribution

In the literature, various approaches have been proposed for data transmission reduction in wireless sensor networks. The state-of-the-art methods can be categorized into four main categories, i.e., data aggregation, data compression, adaptive sampling, and data prediction methods. This section includes a concise review of the existing approaches to data transmission reduction, and focuses on their applications in wearable and body area sensor networks. In this context, the contribution of this paper is explained at the end of this section.

### 2.1. Data Aggregation

Data aggregation methods were designed for multi-hop transmissions, where intermediate nodes can merge several messages received from neighbouring sensor nodes, and send them towards the sink node as a single packet [7,8,9]. As a result, the number of packets transmitted in the network is reduced. Moreover, in case when the sensor nodes are densely deployed, and can observe the same phenomenon, the aggregation methods allow the intermediate nodes to eliminate redundant messages. This approach increases transmission delay as the data have to be buffered before aggregation and transmission, when the intermediate node receives messages from different sources.

In [10] a data aggregation method was introduced to reduce power consumption in wearable sensor networks for physical movement monitoring and quality evaluation of postural control system during walking. The development of the method was motivated by the fact that larger packets have a lower energy consumption per bit. Based on this observation, a routing protocol was introduced, which finds effective routes for data aggregation. The authors have suggested that by transmitting aggregated data though longer paths, a smaller total energy consumption can be achieved than in case of the shortest path communication. However, the longer transmission path results in larger delay. Thus, this approach is not suitable for real-time applications.

A data aggregation algorithm for wearable sensor systems with dynamic topology was proposed in [11]. In that work the wearable sensor networks were considered that connect devices worn by many persons in a group, e.g., a tactical unit. According to that approach the data transmission is based on dynamic routing paths. Each sensor node aggregates data collected by itself and transmitted from its children. The data aggregation algorithm was based on distributed sorting and query scheme that allow every sensor node to send only one message during data transmission round.

Wireless body area networks of smart wearable patches were addressed in [12]. The smart patches can integrate electroencephalography sensors, temperature sensors, electrocardiography sensors, pressure sensors, etc. It was shown that the data aggregation concept can be implemented to combine data from multiple smart patches and transmit them to a base station with reduced energy consumption. The method assumes that nodes are switched over between active and sleep states. The intermediate node in this scheme aggregates data from the sensor nodes that are currently active.

### 2.2. Data Compression

In order to reduce the amount of data transmitted in wireless sensor network, the collected data can be compressed before transmission. Data compression can be performed directly at sensor nodes or at intermediate nodes (cluster heads) [13,14,15]. The compression is usually more effective, when implemented at the cluster heads that receives data from multiple sensor nodes. The data are collected by cluster heads over a period of time, thus the spatial and temporal correlation can be exploited in the compression process. A decompression has to be executed at sink (or base station) to recover the original data.

The compression-based approach leads to delay in collecting data at the sink node. A delay is related to the fact that the cluster head has to hold the data before a data block is accumulated and ready for compression. This approach can also affect the accuracy of collected data, if a lossy compression method is used.

A data compression method for body area sensor network was presented in [16]. The method was designed by taking into account the possibility of overhearing transmissions between the sensor nodes that are attached to human body. It was also observed that the compression is facilitated by strong temporal and spatial correlations among accelerometer readings collected during movement of the body. According to that method, each sensor node samples its data, overhears transmissions of neighbouring nodes, compresses the data, and transmits them. The temporal and spatial correlations were modelled by differential coding and linear regression. An offline procedure was introduced to learn these correlations and adjust model parameters.

In [17] a wearable sensor network was presented for monitoring and quantitative assessment of stroke patients’ upper limb motor function. An accelerometer data compression was implemented in that network to reduce the amount of data during sampling and transmissions. It was shown that raw accelerometer signals can be compressed and precisely reconstructed by the sink node for automatic classification.

The possibility of compressing continuous bio-signals in a wearable sensor network was analysed in [18]. The authors have used a binary permuted block diagonal matrix encoder to compress electrocardiogram and photoplethysmogram data. In that method, the sink node dynamically determines compression parameters to adapt to changing sparsity level of the monitored signal, and transmits the parameters to sensor node.

Another method for electrocardiogram data compression was based on empirical mode decomposition and feature dictionary construction [19]. This solution has demonstrated that the dedicated compression algorithms can achieve higher compression ratio by exploiting self-similarities and inherent properties of the monitored signals. That method was designed for compressing the data transmitted from a single wearable node.

### 2.3. Adaptive Sampling

Adaptive sampling methods allow sensor nodes to adapt their sampling frequency to characteristics of the monitored signal. As a result, they reduce the amount of collected data, decrease number of transmissions and save energy of sensor nodes. According to this approach, the optimal sampling frequency (the time interval between two consecutive samples) is computed online and adapted the sampling to the evolving dynamics of the monitored process [20,21,22]. The adaptation of sampling frequency can be performed collectively for a group of sensor nodes, or individually for each node.

For individual sensor nodes the temporal sampling scheme was proposed based on send-on-delta concept [23]. According to that concept, the data sampling is triggered if the signal deviates by delta defined as the significant change of its value in relation to the most recent sample. In that case, the sensor node does not transmit data readings until they remain within a certain error bound. A dynamic selection of appropriate delta value for the send-on-delta sampling strategy was presented in [24]. That algorithm calculates the delta value for a desired mean transmission rate in real time.

A real time algorithm for selecting the sampling frequency and reducing the amount of data collected by body sensors was discussed in [25]. The adaptive sampling method was developed by using a neural network to predict the subsequent samples and evaluate their uncertainties. According to that approach, a data reading is carried out by a sensor when the uncertainty of prediction is above a predetermined threshold. It was demonstrated that the method reduces the number of measured samples, but preserves the useful high frequency information contained in the original biomedical signals. That method was implemented for measurements of electromyogram, electrocardiogram, electroencephalogram, and acceleration of body parts.

The adaptive sensing approach was also used for extending lifetime of a human activity recognition system with accelerometers [26]. In that case, the trade-off between energy saving and accuracy of the activity recognition was maintained by implementing a feedback control algorithm. The idea underlying that algorithm was to use the measured acceleration data as a feedback to adjust the sampling frequency in each sensor node. It was shown that the optimal sensing frequency depends on position of sensor node and activity type.

In [27] a method was reported for adaptive sampling of electrocardiogram. That method adjusts the sampling frequency by taking into account a medical relevance of the data. The data relevance was evaluated using eye tracking technology. Thus, that method generalises the knowledge from cardiology expert perception of the electrocardiogram. The main objective of that approach was to ensure a good reproducibility of the diagnostic features that are necessary for interpretation of the collected data by experts.

### 2.4. Data Prediction

Prediction-based approach enables selective transmission of the data collected by sensor nodes [28,29,30]. According to this approach, only a subset of the collected data is delivered to the sink node. Each sensor node has to decide whether to send its current data readings to the sink. When data are not transmitted, the sink has to reconstruct them. To this end, the same prediction algorithm is used at the sensor node and at the sink for estimation of the current data readings. If the prediction error is above a predetermined threshold then the sensor node sends its data readings to the sink. In opposite situation, the data are not transmitted and the sink uses the prediction as an accurate estimate of the sensed data.

A linear prediction algorithm was used in [31] to predict sensor values and transmit the sensor data if the difference between the predicted value and the real sensor reading exceeds a predetermined limit. Simulation experiments have revealed that such approach reduces the number of transmission effectively for signals representing typical control system responses, i.e., step response of a first-order system and step responses of secondorder systems.

The prediction-based method of data transmission reduction was exploited by several authors to address the problem of energy conservation in body area sensor networks. In [32] a generic prediction algorithm was proposed for predicting the data sampled by wireless body sensors on the basis of the proportional–integral–derivative (PID) control technique. That algorithm was designed for monitoring of hemodynamic and electrocardiographic signals. A similar method with application of artificial neural network was presented in [33]. The artificial neural network was used for prediction of physiological data (blood pressure and electrocardiogram) collected by wireless body sensors.

Recently, the prediction-based data transmission concept was adapted for air quality sensors [34]. To this end, a prediction algorithm was proposed, which uses a wavelet transform and Gaussian processes model. That algorithm transforms the sensor readings to the wavelet domain and then the Gaussian processes prediction is made for each subband of the wavelet transform. It was shown that the multi-resolution analysis can improve the prediction results for complex series of sensor readings.

### 2.5. Other Methods

Apart from the methods dedicated for sensor networks, many interesting solutions have been also proposed to reduce data transmissions and extend battery lifetime of individual wireless sensor nodes, Internet-of-Things (IoT) devices, and networked control systems.

For instance, energy savings can be achieved in wearable devices, by eliminating unnecessary data processing operations. In [35] an event-triggered machine learning approach was introduced, which enables the sensor node to perform feature extraction only when it is necessary for accurate fall detection. According to that method, an event is detected if magnitude of accelerometer readings is above a threshold. The detected event triggers feature extraction and classification procedures. It was shown that such approach improves the accuracy of falls detection. However, that study has considered only two types of human activity (falls, and non-falls).

In [36] a method was proposed for IoT devices to enable energy-aware sampling of GPS signal and processing of location data. That method utilizes cognitive control and event recognition techniques. The proposed cognitive control algorithm reduces uncertainty about user mobility by adapting a sampling policy to current state of mobility. The adaptive location sampling is implemented only when relevant events are detected.

An event-based networked control systems was presented in [37]. In such system the limited bandwidth of wireless network has to be effectively used for transmitting state and control information. Thus, the data transmissions among sensors, controller and actuators are performed when difference between sensor signal and a reference signal is above an event threshold.

Other approaches to reduction of transmissions and energy consumption in wireless sensor networks are based on appropriate design of medium access control (MAC) protocols. A number of the MAC protocols for wireless body area networks have been analysed in [38]. The analysis took under consideration reliability, delay, collision and energy consumption. It should be noted that the method presented in this paper can be integrated with the MAC layer or routing layer transmission reduction for further energy savings.

### 2.6. Our Contribution

The proposed approach is based on a different concept than the above-discussed state-of-the-art methods. According to this approach, the sensor nodes use a classifier to decide whether the data readings have to be transmitted. The classifier is trained in advance to select the useful data, which are necessary for a target application of the wearable sensor network, e.g., for recognition of human activities.

The state-of-the-art methods are aimed at transmitting the reduced data, which allow the receiver node to reconstruct the original series of sensor readings. In contrast, the new method presented in this paper is designed to transmit the selected data that are sufficient for the receiver node to made accurate decisions with use of embedded machine learning algorithms. The motivation for this work was that in many situations only a subset of the data from sensor nodes is required by the machine learning algorithms to make correct decisions. For instance, the activity or position of a monitored person in some cases can be recognized based on data from a single wearable sensor, while in other circumstances the data from several nodes are necessary for correct recognition.

Unlike the data aggregation and data compression approaches, the proposed method does not involve the buffering of sensor readings before transmission, which results in additional delay. Thus, the presented solution is more suitable for real-time applications. Moreover, our method is suitable for networks with different topologies, including those without multi-hop transmissions.

The contribution of this work also includes experimental evaluation of the transmission reduction methods with use of real wireless wearable sensor network. Comparison of the proposed method, with prediction-based approaches, was conducted for physical prototypes of wearable nodes. Results of this work include analysis of the wearable sensor network lifetime in real-world application. In contrast, the results presented in many previous works were obtained with use of simulation techniques.

The main contributions of this paper are summarized as follows:

- A new concept using embedded classifiers for data transmission reduction in sensor networks is presented. According to the best authors’ knowledge the embedded machine learning algorithms have not been used so far in this context. Existing works are limited to simpler case, where individual IoT devices are considered.
- An algorithm is introduced for preparing a data set, which facilitate training of the classifiers designed to eliminate unnecessary data transmissions.
- Feasibility and effectiveness of the proposed approach was confirmed in experiments with wearable sensor network for human activity monitoring.

## 3. Proposed Method

### 3.1. Overview of the Method

The objective of the considered wireless sensor network is to recognize human activities $a_{t}$ in successive time steps (*t*). In this network, the wearable sensor nodes attached to a person establish a cluster, where one of the nodes acts as cluster head, i.e., receives data from the other nodes and uses them to recognize the user’s activity. The recognized human activity is periodically reported by the cluster head to a sink node.

The method presented in this section allows us to reduce the number of data transmissions from sensor nodes to cluster head. According to this method, the sensor nodes use classifiers to decide if sensor data are necessary for activity recognition and have to be transmitted, or not. The decision made by sensor node *i* with use of binary classifier *C* can be described with the following formula:

(1) $$d_{i,t}=C\left(S_{i,t},M_{i}\right),$$

where $d_{i,t}=1$ means that the data collected at time step *t* by sensor node *i* have to be transmitted to cluster head and $d_{i,t}=0$ denotes that the data transmission can be skipped. This decision is made by the classification algorithm *C*, based on data set $S_{i,t}$ and model $M_{i}$. The models $M_{i}$ are trained using a machine learning algorithm, according to the procedure which is presented in Section 3.3. The set $S_{i,t}$ consists of preprocessed sensor readings collected by sensor node *i* at time step *t*. Construction of the classification algorithm *C* depends on the machine learning algorithm used for training the model $M_{i}$. For instance, if the model is trained with use of the random forest algorithm then it has the form of a collection of decision trees, and algorithm *C* has to calculate the decision based on those decision trees. The operations performed by sensor node are summarized in Algorithm 1.

It should be noted that the method described in this section can be implemented for various sets of sensors, with use of different machine learning algorithms. Here, for seek of generality, the term “machine learning algorithm” is used, which can refer to neural network, decision tree, random forest, support vector machine, etc. Similarly, the data set mentioned here can be composed of different sensor readings. Thus, size of the processed and transmitted data is not determined. Implementation details of the proposed method, for a prototype of wearable sensor network, are discussed in Section 4.
**Algorithm 1** Operation of sensor node *i* **Input:**classification model $M_{i}$1:**for** each time step *t* **do**2:    collect data $S_{i,t}$ from own sensors3:    classify collected data-determine $d_{i,t}=C\left(S_{i,t},M_{i}\right)$4:    **if** $d_{i,t}=1$ **then**5:        send $S_{i,t}$ to cluster head6:    **end if**7:**end for**

As shown in Algorithm 2, the cluster head (node $i^{*}$) collects its own sensor readings and waits for data transmitted from the other nodes in its cluster. The available data are then used to recognize current activity of the monitored person. Since the proposed method assumes that only selected data are sent to the cluster head node, an activity recognition algorithm *R* is needed, which can deal with incomplete data sets. In general, the activity recognition task, performed by cluster head, is expressed as follows:

(2) $${\hat{a}}_{t}=R\left(S_{t},M\right),$$

where *M* is an activity recognition model trained with use of a machine learning algorithm and $S_{t}$ denotes a set of data transmitted to the cluster head from sensor nodes a time step *t*:

(3) $$S_{t}=\left\{S_{i,t}:d_{i,t}=1\right\}.$$

The problem of reducing data transmission at time step *t* is defined as follows:

(4) $$\begin{array}{cccc} \; & \mathrm{minimize}\; & \; & \mathrm{card}(S_{t})\\ \; & \mathrm{subject}\;\mathrm{to}\; & \; & {\hat{a}}_{t}=a_{t}, \end{array}$$

where $a_{t}$ is the actual human activity, and card(·) denotes cardinality of the set. Note that card($S_{t}$) corresponds to the number of sensor nodes that have to send data at time step *t*.
**Algorithm 2** Operation of cluster head (node $i^{*}$) **Input:**recognition model *M*1:**for** each time step *t* **do**2:    collect data $S_{i^{*},t}$ from own sensors3:    wait for data $S_{t}$ transmitted from sensor nodes4:    recognize activity ${\hat{a}}_{t}=R\left(S_{i^{*},t}\cup S_{t},M\right)$5:    send ${\hat{a}}_{t}$ to sink6:**end for**

The classification models $M_{i}$, should allow sensor nodes to made decisions $d_{i,t}$ that determine solution of minimization problem (4). This objective is achieved by appropriate training of the classification models. Application of this proposed method involves the following steps:

1. Collect training data.
2. Divide the training data into two samples.
3. Train recognition model *M* using the first data sample.
4. Prepare data for training classification models $M_{i}$ based on the second data sample.
5. Train classification models $M_{i}$.

A training data set, collected at the first step, has to include preprocessed sensor readings from all sensor nodes (*S*) and information about activities of the monitored persons (*A*) for a representative period. Activities are recognized by a human observer. The sensor readings are prepossessed to eliminate noise, aggregate the raw data and reduce their size. Subsequently, the training data set (S,A) is divided into samples ($S^{\prime },A^{\prime }$) and (S″,A″). Finally, the training procedures (steps 3–5) are performed as discussed in the following subsections.

**Figure 1:**
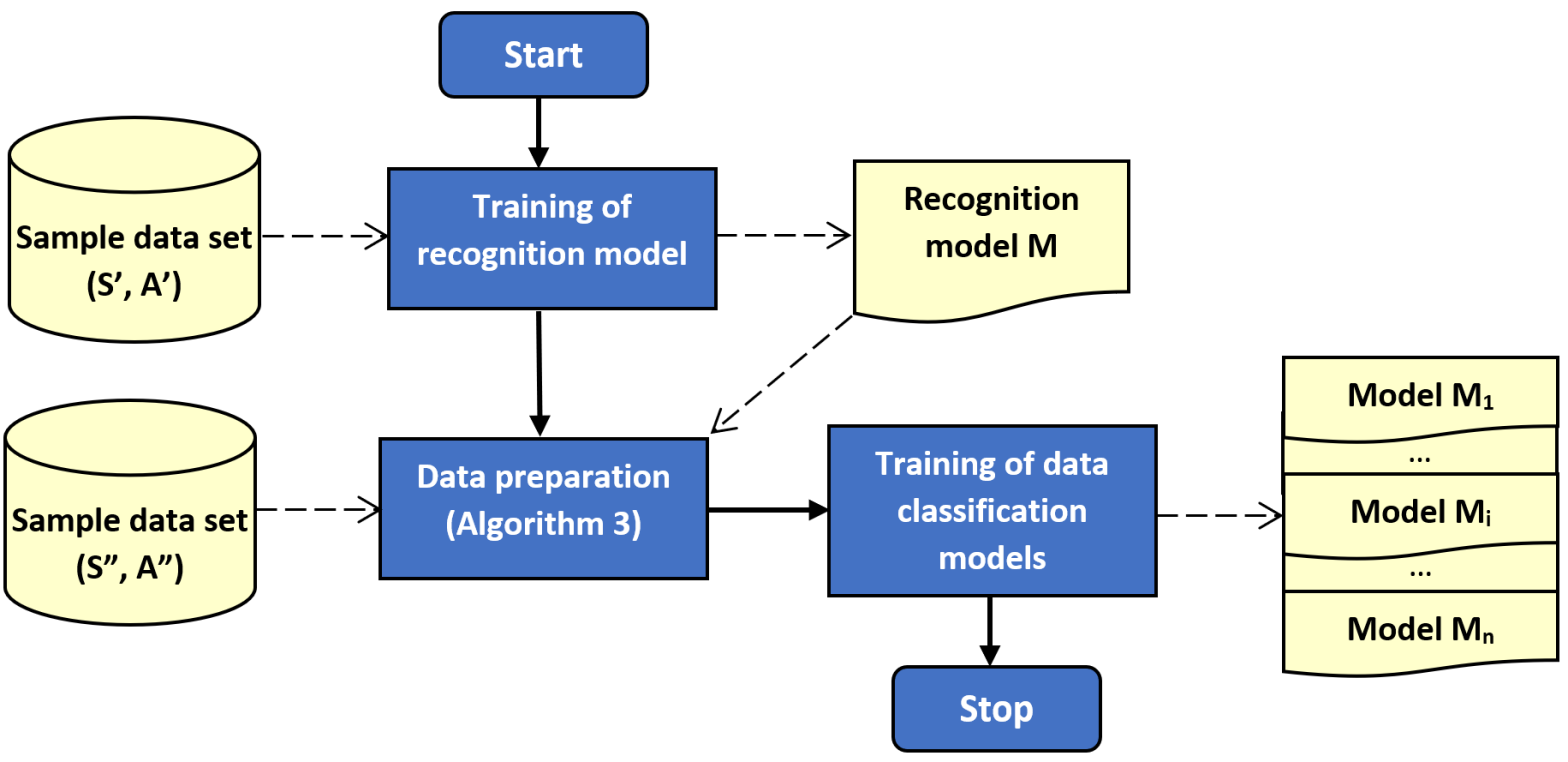
Block diagram of the proposed method: training stage.

**Figure 2:**
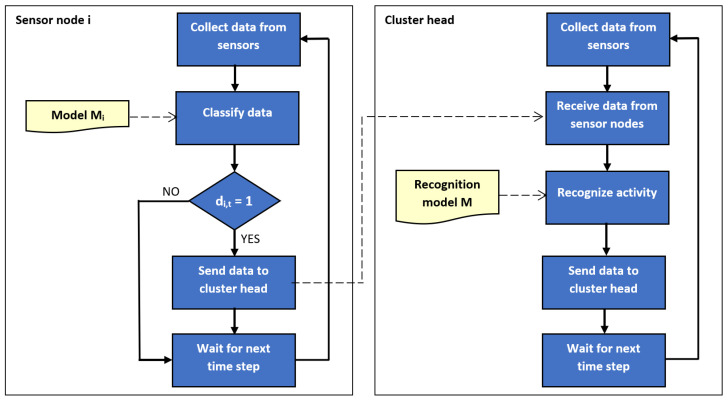
Block diagram of the proposed method: network operation stage.

The outline of the proposed method is illustrated by block diagrams in Figure 1 and Figure 2. Figure 1 depicts the training of recognition and classification models. Usage of the trained models during operation of sensor network is presented in Figure 2. It should be noted that left part of Figure 2 corresponds to Algorithm 1, and the right part relate to Algorithm 2.

### 3.2. Activity Recognition Model

A model *M* for activity recognition is created using machine learning algorithms. As it was already mentioned above in this section, the human activity has to be recognized based on incomplete data sets. In order to deal with the missing (not transmitted) data, an ensemble learning method was used in this study.

To be specific, the considered recognition model *M* is an ensemble of sub-models. Each sub-model is trained to recognize human activity based on data from different subset of the sensor nodes. For instance, let us consider a network composed of cluster head and one sensor node. In this case two sub-models are used. The first sub-model, which takes into account data from both nodes, will recognize the activity when data from the sensor node are transmitted. In opposite situation, the second sub-model will recognize the activity on the basis of data only from the cluster head.

Hereinafter, the symbol SM(Z) stands for the sub-model, which is designed to recognize the human activity based on data delivered from sensor nodes belonging to subset *Z*. A separate sub-model SM(Z) is trained for each non empty subset of nodes *Z*, such that Z⊆I, where I={1⋯n} denotes a set of indices used for identification of sensor nodes, and *n* is the total number of sensor nodes. The sub-models training is based on data sample ($S^{\prime },A^{\prime }$), which includes examples of sensor readings ($S_{i,t},i\in I,t\in T$) and labels indicating relevant human activities ($a_{t},t\in T$) for time period *T*. The examples used for training of sub-model SM(Z) are obtained from ($S^{\prime },A^{\prime }$) by selecting the sensor readings for the nodes that belong to *Z*. These examples are defined as tuples $\left(S_{t},a_{t}\right)$, where $S_{t}=\left\{S_{i,t}:i\in Z\right\}$ and t∈T.

The number of all possible sub-models equals to $2^{n}-1$. Thus, this number increases fast with the number of sensor nodes (*n*). However, the considered wearable sensor networks for human activity monitoring consist of only few sensor nodes [32] and in this case the above model ensemble can be easily implemented (e.g., for n=3 the number of all sub-models equals 7). In order to recognize human activity, during operation of the sensor network, the cluster head selects appropriate sub-model SM(Z), where *Z* corresponds to the set of senor nodes that currently deliver data readings. It should be noted that the cluster head node always has the access to its own sensor readings. Thus, if $i^{*}$ is the identifier of cluster head then the ensemble model needs to include sub-models SM(Z), where $i^{*}\in Z$. This observation allows us to reduce the number of sub-models that have to be stored by the sensor node $i^{*}$, as the sub-models SM(Z), where $i^{*}\notin Z$ can be omitted. Thus, the number of necessary sub-models is reduced to $2^{n-1}$, i.e., for n=3 we need 4 sub-models.

### 3.3. Models for Sensor Data Classification

The classification models $M_{i}$ are used to decide if the data collected by sensor node *i* at time step *t* have to be transmitted or not. In order to train the classification models $M_{i}$ it is necessary to collect examples of optimal decisions $d_{i,t}$. These decisions are determined by solving the minimization problem (4) for the sample data set (S″,A″), as presented in Algorithm 3.

The input of Algorithm 3 includes preprocessed data readings from all sensor nodes ($S_{i,t}$) and labels representing actual human activities observed during collection of the sensor readings ($a_{t}$). Moreover, Algorithm 3 requires the activity recognition model *M*, which has to be initially trained on a separate data set, as discussed in Section 3.2. For each time instance (*t*) in the sample data set, the algorithm finds the smallest subset of sensor nodes (*Z*) that includes the cluster head node $i^{*}$ and enables correct recognition of activity $a_{t}$.

When analysing the pseudo-code of Algorithm 3, it should be noted that this algorithm starts the search with $Z=i^{*}$, when k=0 and X=∅. It means that at the first step it is checked whether the data collected solely by cluster head are sufficient for recognizing the human activity. If not, then the algorithm tries to made the recognition based on data from cluster head and one additional sensor node (k=1). The additional sensor node is selected randomly and all possible pairs of nodes are verified. During subsequent iterations, the number of utilized nodes is increased, until the human activity is correctly recognized or all nodes in the cluster are taken into account.

The resulting subset of sensor nodes is identified by binary variables $d_{i,t},i=1\cdots n$, where *n* corresponds to the total number of sensor nodes and meaning of the $d_{i,t}$ values was explained above for Equation (1).

As discussed in Section 3.1, separate classification model $M_{i}$ is needed for each sensor node ($i\neq i^{*}$) to make decisions about necessity of data transmissions (see Algorithm 1). The training of models $M_{i}$ is performed with use of sensor readings from the sample data set (S″) and decisions *D* determined by Algorithm 3. Formally, the set of data used to train model $M_{i}$ for *i*-th sensor node is represented as a set of tuples $\left(S_{i,t},d_{i,t}\right)$, where t=1⋯,m.
**Algorithm 3** Data preparation for training of models $M_{i}$ **Input:**$S\prime \prime =\{S_{i,t}:i=1\cdots n,t=1\cdots m\}$, $A\prime \prime =\{a_{t}:t=1\cdots m\}$, *M*, $i^{*}$ **Output:**$D=\{d_{i,t}:i=1\cdots n,t=1\cdots m\}$1:**for**t=1⋯m**do**2:    found= false3:    k=04:    **repeat**5:        X= set of all *k*-element subsets of $\left\{\left\{1,2,\cdots ,n\right\}\setminus \left\{i^{*}\right\}\right\}$6:        **repeat**7:           Z= random element from *X*8:           X=X∖Z9:           $Z=Z\cup \{i^{*}\}$10:           $S_{t}=\left\{S_{i,t}:i\in Z\right\}$11:           **if** $R\left(S_{t},M\right)=a_{t}$ **then**12:               found= true13:           **end if**14:        **until** found **or** X=∅15:        k=k+116:    **until** found **or** k=n−117:    **for** i=1⋯n **do**18:        **if** found **and** i∉Z **then**19:           $d_{i,t}=0$20:        **else**21:           $d_{i,t}=1$22:        **end if**23:    **end for**24:**end for**

Algorithm 3 searches for the smallest subset of sensor nodes that enables correct recognition of the human activity. This search is repeated for each time step covered in the sample data set. Dominant operation in this algorithm is the test of activity recognition for a selected subset of nodes (line 11). Each considered subset of sensor nodes has to include the cluster head node, thus the total number of possible subsets equals $2^{n-1}-1$. In the optimistic case, data collected by the cluster head are sufficient for correct recognition, and only one test is performed. The pessimistic case is that the test is executed for all possible subsets. On average, $(2^{n-1}-1)/2$ tests are required. It means that the average time complexity of the algorithm is $O(m\left(2^{n-2}-0.5\right))$. It should be kept in mind that this algorithm is used at the training stage, and its execution time does not impact the operation of sensor network.

## 4. Experiments

The objective of the conducted experiments was to verify effectiveness of the proposed method, when implemented in the wearable sensor network for human activity monitoring. The activity recognition was considered as a typical application of the wearable sensors. During experiments, the energy consumption of sensor nodes was measured to enable evaluation of the network lifetime. In parallel, the accuracy of activity recognition was investigated. The results obtained for the proposed method were compared with those of the state-of-the-art data prediction methods that use neural networks [30] and naive algorithm [39].

### 4.1. Experimental Testbed

**Figure 3:**
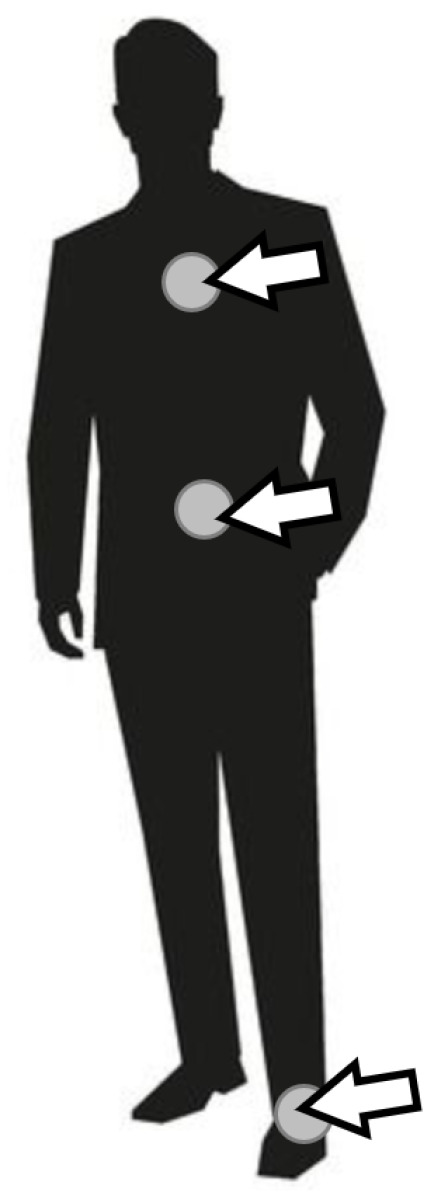
Placement of sensor nodes.

The prototype of wearable sensor network used for the experiments was built of four sensor nodes. Each node contains an ARM microcontroller (STM32F103C8T6) and communication module based on ZigBee technology (xBee S2C). Three of them are additionally equipped with the accelerometer (MPU-9250) and the light sensor (ALS-PT19). These sensor nodes are used as the wearable devices attached to a person, as shown in Figure 3. The fourth node acts as the sink and collects the information about recognized activities of the monitored person.

Selection of the sensors for the experimental prototype was based on literature review. The accelerometers were used to enable recognition of human activities, such as sitting and walking. This type of accelerometer application is popular in the literature [40,41,42]. Additionally, the light sensors were included in the wearable nodes to enable coarse localization of the person and detection of active computer monitors. Results reported in previous works have confirmed that the light sensors can be useful for indoor localization [43,44].

In order to measure energy consumption, the LTC4150 Coulomb counter was connected to each sensor node. The Coulomb counter enables estimation of residual battery energy in wireless devices. Its operation is based on differential measurement over a shunt, i.e., a small resistance connected in series. The Coulomb counter measures voltage drop across the resistor and evaluates current flow in the circuit using Ohm’s law. The smallest amount of energy, which can be measured equals 26 µAh. This energy results from the parameters of the internal resistor and the maximum energy consumption of the sensor node. The LTC4150 module provides a signal on an output each time the energy of 0.1707 mAh is consumed by the sensor node. These signals were counted during experiments by microcontroller of the sink node to determine the energy consumption over a long period of time.

It was assumed that all sensor nodes have the same battery capacity of 700 mAh. A balance between energy consumption of individual sensor nodes was achieved by implementing the cluster head rotation algorithm presented in [45]. Death of sensor node was detected each time the sensor node has consumed the predetermined amount of energy. The network lifetime can be determined with the different assumptions described in the previous section. The network lifetime was determined in this study as the time from the start of sensor network operation to the discharge of one of sensor nodes. This is due to the significant drop in recognition accuracy after any of the wearable nodes is discharged.

**Figure 4:**
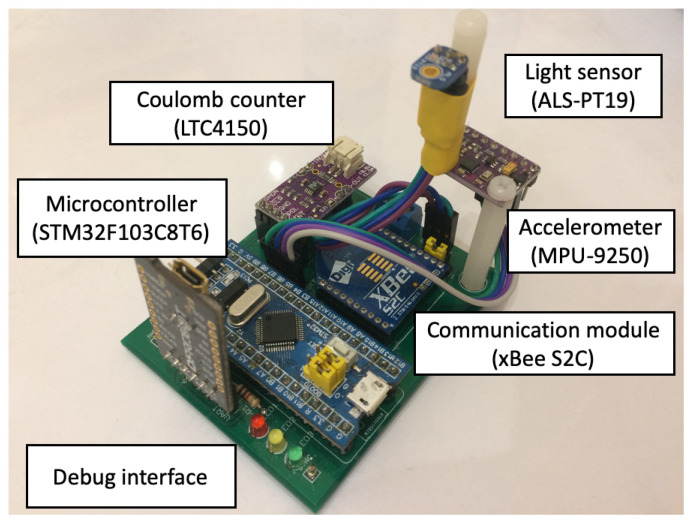
Sensor node.

**Figure 5:**
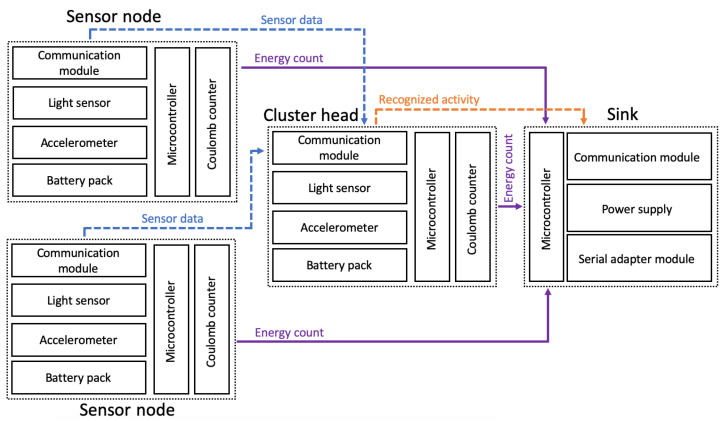
Block diagram of the experimental testbed.

The prototype of single wireless sensor node with connected Coulomb counter is presented in Figure 4. Block diagram of the testbed used in experiments with wireless wearable sensor network was shown on Figure 5. At the training stage, all data readings of accelerometers and light sensors were preprocessed by the sensor nodes (as explained later in this section), collected by the sink, and transferred to a workstation. In this way the sample data set was gathered for training the classification and recognition models. The models were trained on the workstation with use of Waikato Environment for Knowledge Analysis (WEKA package) and Konstanz Information Miner (KNIME) software [46]. The KNIME allows us to use both KNIME machine learning implementations and the WEKA repository that gives over 100 various methods and their variants. It proved to be useful in data exploration and finding machine learning solutions in previous works. For a small number of sensor nodes, the experiments could be conducted in a reasonable time using KNIME. For larger networks, Python or MATLAB would be more useful. The KNIME workflow containing the data set is available at http://biometrics.us.edu.pl/request/database/activity. Based on initial experiments, the random forest algorithm was selected. This algorithm, together with the trained models, was deployed on the sensor nodes.

**Figure 6:**
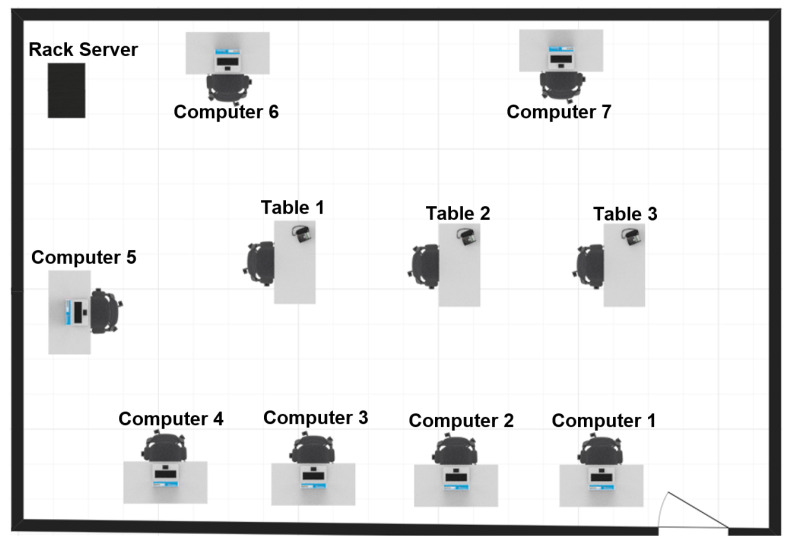
Computer laboratory.

The developed prototype of wearable sensor network was used to recognize human activity in a computer laboratory. A layout of the computer laboratory is shown in Figure 6. Only artificial light was available in the laboratory during experiments. The set of recognized human activities includes 19 cases: walking in the laboratory, standing by the rack server, sitting by table 1, 2, or 3, sitting by computer 1–7, and working on computer 1–7. The difference between the cases of sitting by a computer and working on a computer is that the working person uses keyboard, and the monitor is switched on.

The prototype of wearable sensor network collects sensor readings from accelerometers and light sensors in time steps of one second. At each time step, after registering new data, the sensor nodes calculate mean, standard deviation, 10-th percentile, and 90-th percentile, based on the last collected 15 sensor readings. The number of 15 sensor readings used for these calculations was selected empirically. When more readings are taken into account then the noise is better eliminated. However, at the same time, some short-term changes of activity can be not detected. Thus, we have chosen the smallest number of readings that allows us to achieve accurate recognition of the human activities.

The calculations of mean, standard deviation, and percentiles are performed for the measured light intensity and magnitude of acceleration. Based on collected data, the activity recognition is performed by cluster head, and the result is reported to the sink every second.

### 4.2. Results and Discussion

**Figure 7:**
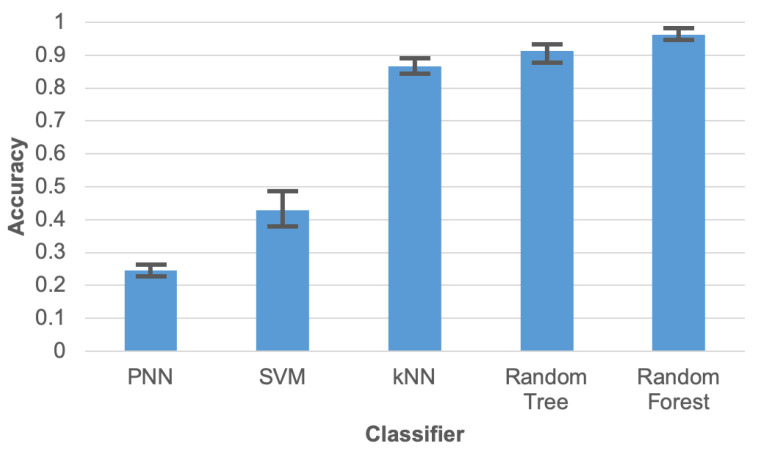
Accuracy of human activity recognition for compared machine learning algorithms.

Initial experiments were conducted to compare the accuracy of human activity recognition for various machine learning algorithms (classifiers). The compared algorithms include: probabilistic neural network (PNN), support vector machines (SVM), k-nearest neighbours algorithm (k-NN), random tree (RT), and random forest (RF). During these tests the data reduction was not executed, i.e., all collected sensor readings were taken into account. The initial tests were carried out using implementations of the machine learning algorithms that are available in the WEKA package. Results of this comparison (Figure 7) clearly demonstrate that the data collected by the proposed wearable sensor network enables recognizing the human activities with a high accuracy. Parameters of the compared machine learning algorithms were selected empirically based on our preliminary experiments.

During experiments 60% of the data were used for training and 40% for testing. The test was executed 10 times for different divisions of the data into train and test sets. The error bars presented in Figure 7 correspond to maximum and minimum results of these tests, while columns depicts the average value.

The PNN was trained using the constructive training algorithm, based on dynamic decay adjustment [47]. When using this algorithm, the PNN is dynamically constructed during training and the number of required hidden neurons is automatically adjusted. The PNN is built of neurons with a Gaussian activation function and models the probability distribution of each considered class through a combination of these Gaussians. The training algorithm adjusts each Gaussian function by taking into account two parameters ($\Theta^{+}$ and $\Theta^{-}$) to avoid conflicts between different classes. The parameter settings used in this study are $\Theta^{+}=0.4$ and $\Theta^{-}=0.2$.

The training of SVM aims at constructing hyperplanes defined in a multidimensional space that separate training data points belonging to different classes. To this end a training algorithm is used, which solves an optimization problem [48]. The iterative training procedure finds optimal hyperplanes with maximum distance to the nearest training data point of any class. The experiments were performed using C-SVC version of the SVM classifier [49] with radial basis function kernel.

The k-NN classifier [50] evaluates distances between a test data point and all training data points in a multidimensional feature space. Based on the calculated distances, the nearest *k* training data points are selected. The classification result is determined as the class, which is most common among the *k* selected training data points. This algorithm was used during experiments with parameter k=3, and the Euclidean distance was considered.

The RT algorithm [51] builds a decision tree using a random procedure. Each node of the tree is split using the best split among a subset of randomly chosen attributes. The tree constructed by the RT algorithm considers *K* random attributes at each node. In this study the parameter K=5 was used.

The highest accuracy was achieved for the RF algorithm. Thus, this algorithm was selected for further experiments. The RF algorithm [52] creates a set of decision trees from randomly selected parts of training data and features. Each of the multiple decision trees classifies data independently and votes for the selected class. Finally, the votes from all decision trees are aggregated to decide the output class. The classification algorithm picks the class having the majority of votes from decision trees.

**Figure 8:**
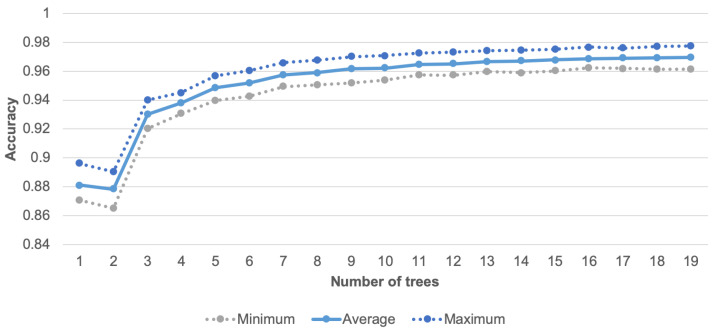
Accuracy of human activity recognition for different number of trees.

The number of trees for RF algorithm was set to 10. As illustrated in Figure 8, for more than 10 trees, the increase of recognition accuracy is not significant (below 0.006%), while the larger number of trees involves longer computational time and increased consumption of memory resources. The doted lines in Figure 8 correspond to the maximum and minimum result of 10 tests, while the solid line represents the average value. An additional advantage of the random forest algorithm is its suitability for implementation in the prototypes of sensor nodes. It should be noted that several approaches to implementation of the random forest classifier for embedded devices are available in the literature [53,54,55]. In this study the random forest algorithm was used for activity recognition as well as for data classification to decide which data have to be transmitted.

**Figure 9:**
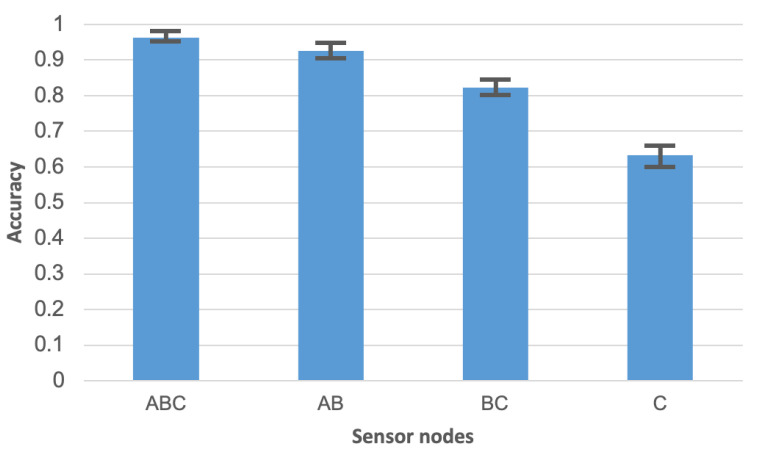
Comparison of activity recognition accuracy for sensor network and separate sensor nodes.

**Figure 10:**
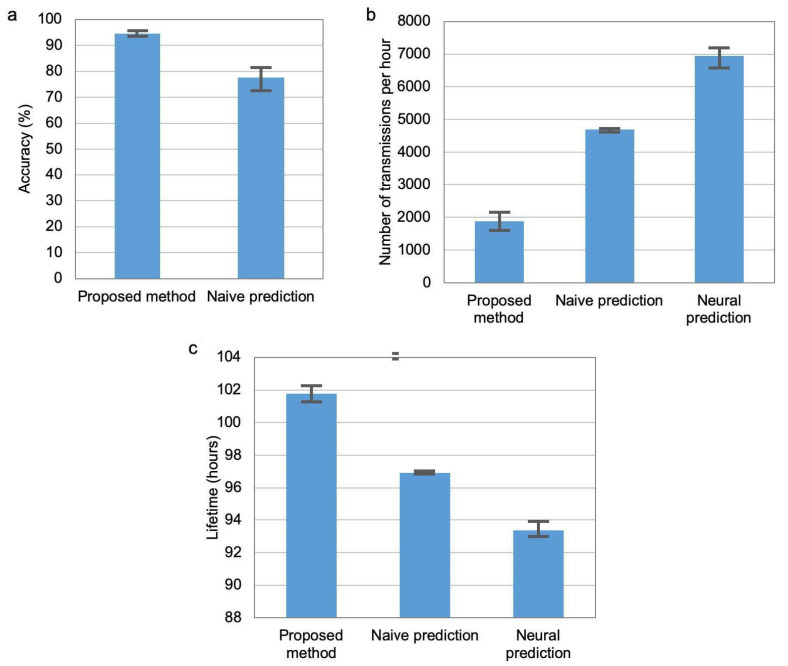
Comparison of accuracy (**a**), transmission number (**b**), and network lifetime (**c**).

A high accuracy of human activity recognition was achieved using the sensor network with three wearable nodes. Figure 9 compares this result with the accuracy obtained in case when the input data for activity recognition algorithm are collected from one sensor node only. It should be noted that the experiments were performed by using the random forest algorithm for activity recognition. The Labels A, B, and C in Figure 9 identify the sensor nodes. ABC denotes the sensor network of three nodes. Sensor node A was placed on the person’s chest, node B on the waist, and node C on the leg. Based on the chart in Figure 9, it can be observed that the accuracy obtained for separate sensor nodes are significantly lower than for the sensor network. This observation confirms that the use of network with three sensor nodes is justified. The meaning of error bars in Figure 9 and Figure 10 is the same as discussed above for Figure 7.

The main experiments were carried out in a time period of three weeks. Figure 10 compares the recognition accuracy, number of transmissions and network lifetime for the three methods of transmission reduction. The chart in (Figure 10a) shows the recognition accuracy, which was achieved when the network lifetime was equal for the compared methods (equal to 101 h and 50 min). It should be noted that the method based on neural prediction did not reach this value of network lifetime, thus it was not considered in the chart. The recognition accuracy for the proposed approach is significantly higher than for the naive prediction method. It should be noted that the columns in Figure 10 represent average results, while error bars correspond to minimal and maximal values of the determined parameters for 10 runs of the experiment.

The results shown in (Figure 10b,c) relates to the situation when the accuracy of activity recognition is at the same level (average of 0.95%) for the three compared methods. Number of data transmissions from sensor nodes to cluster head per hour is analysed in Figure 10b) and network lifetime in Figure 10c). Note that the maximum number of data transmissions from two sensor nodes to cluster head during one hour equals 7200. It can be observed that the proposed method is more effective in reducing data transmission and extending network lifetime than the state-of-the-art methods.

**Figure 11:**
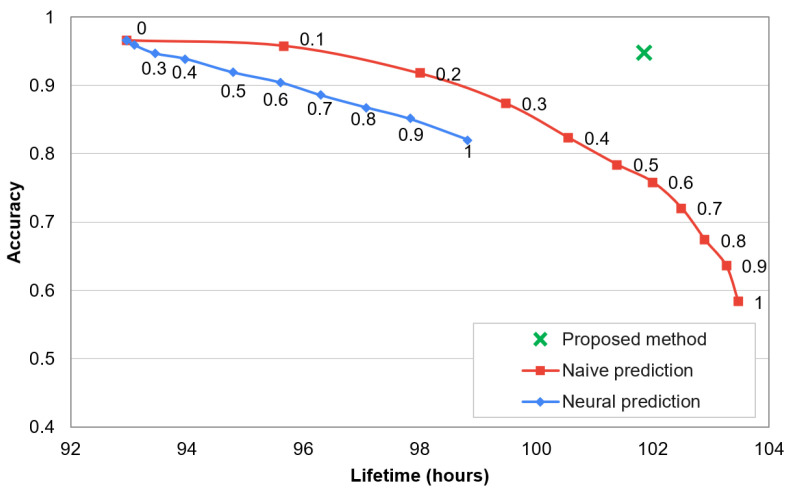
Accuracy of activity recognition vs. lifetime of sensor network.

**Figure 12:**
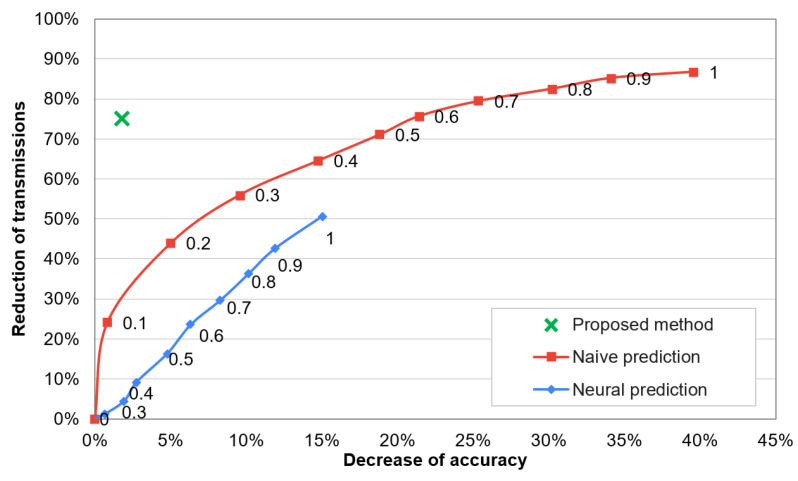
Reduction of transmissions vs. accuracy decrease of activity recognition.

Dependency between recognition accuracy and network lifetime for the compared methods is analysed in Figure 11. The numbers presented near data points in this chart describe threshold values for the state-of-the-art methods. By using the state-of-the-art methods with higher threshold value, the lifetime of sensor network can be extended. However, the recognition accuracy is significantly decreased when extending the lifetime. In contrast, the proposed method enables a significant network lifetime extension and maintains the high recognition accuracy of human activities. The decrease of accuracy and reduction of data transmissions are analysed in Figure 12. In these results it can be observed that the proposed approach decreases the recognition accuracy by only 1.8%, while reduces 75% of data transmissions. The compared algorithms achieve a significantly worse trade-off between the accuracy and data reduction. The reason behind the superior effectiveness of the proposed approach is that the selection of transmitted data is well fitted to the needs of the recognition algorithm. This fitting is achieved by appropriate training of the classifiers that control the selection of transmitted data.

It should be noted that only one data point is presented for the proposed method in Figure 11 and Figure 12 because this method does not use a threshold parameter. The compared prediction-based methods assume that some error of the collected data is acceptable. This acceptable error level is expressed by the threshold parameter. In contrast, the main objective of the proposed method is to maintain the high recognition accuracy when reducing the data transmission. Thus, a parameter (threshold) that would allow us to intentionally decrease the accuracy is not introduced.

**Figure 13:**
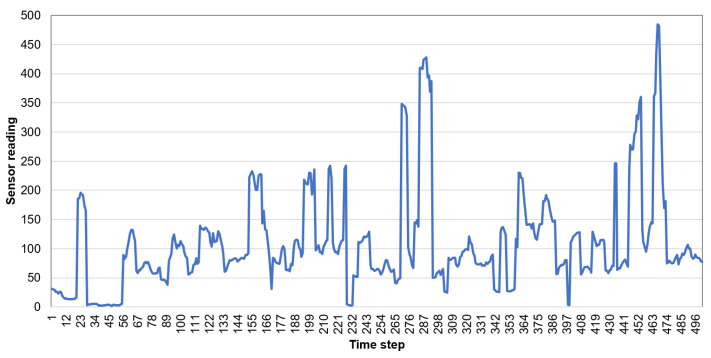
Readings of light sensors collected during walk (mean value for time window).

When comparing the naive and neural prediction methods, it can be observed that the first one provides better results. The reason is that the naive algorithm more accurately predicts the chaotic time series of sensor readings. The chaotic character of the sensor readings collected by wearable devices during walk is illustrated by the example of light sensor data in Figure 13.

**Figure 14:**
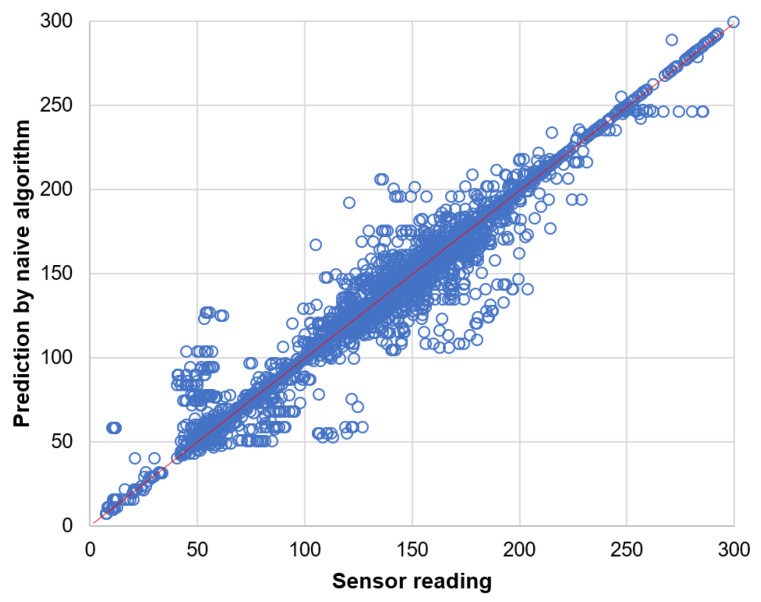
Prediction of light sensor readings by naive algorithm.

**Figure 15:**
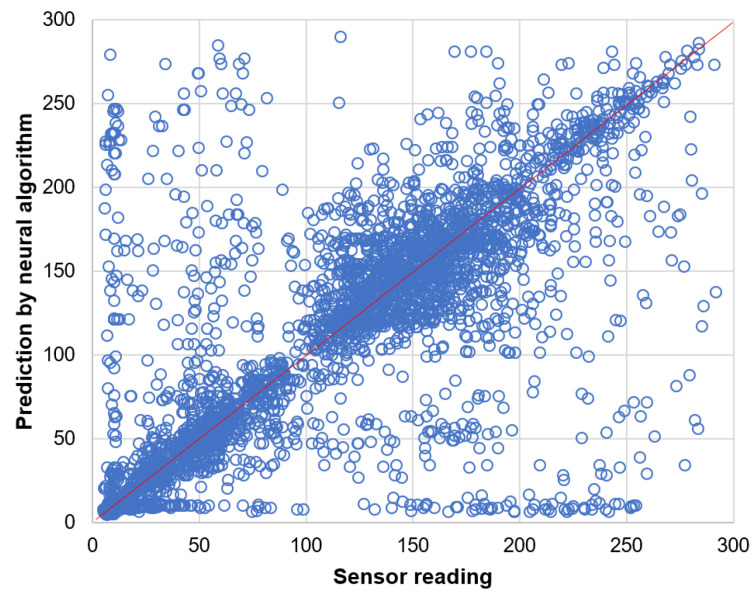
Prediction of light sensor readings by neural algorithm.

Prediction results for the data readings of light sensor are presented by the scatter plots in Figure 14 and Figure 15. These results clearly show that the naive prediction (Figure 14) is more accurate than the neural network (Figure 15) as the data points in scatter plot for naive prediction are more closely concentrated along the diagonal line.

## 5. Conclusions and Future Work

The experimental results presented in this paper confirm that lifetime of the activity recognition system with wearable sensor network can be significantly extended by using the embedded classifiers that detect useful sensor readings. The introduced approach allows us to effectively eliminate the data transmission that are not necessary for performing the given recognition task. This reduction of data transmission preserves high recognition accuracy. In this study, the presented method was applied for human activity recognition, however it can be easily adapted for different applications of wearable sensor networks that involve the use of recognition algorithms for other purposes. It is also suitable for application in Internet-of-Things environment. Comparison with state-of-the-art solutions have shown that the proposed method achieves better trade-off between the accuracy and transmission reduction. 75% of data transmissions from sensor nodes were eliminated, while the accuracy level of 95% was kept.

The model ensemble used in this study for activity recognition is suitable for the wearable sensor network, in which a few nodes are used to monitor the person. Future works will be devoted to other types of the sensor network, where a larger number of sensor nodes is connected in one cluster, and different ensemble learning methods will be used. For instance, a separate recognition sub-model can be trained based on data from each sensor node. In this case, after receiving data from sensor nodes, the recognition is performed independently using many sub-models in parallel. Subsequently, a common decision is made in a voting procedure, by taking into account the recognition results obtained from individual sub-models. This method requires only *n* sub-models to be trained, where *n* corresponds to the number of sensor nodes. Another possibility is to consider group of sensor nodes to simplify the model.

An interesting topic for further research is also the investigation of multi-layered transmission reduction with integration of the proposed method and dedicated MAC layer or network layer protocols.

## Data Availability

Publicly available dataset was analyzed in this study. This data can be found here: http://biometrics.us.edu.pl/request/database/activity.

## Abbreviations

*A*	Time series containing information about observed human activities
$A^{\prime }$	Part of time series *A* used to train the recognition model *M*
A″	Part of time series *A* used to train the classification models $M_{i}$
$a_{t}$	Actual human activity at time step *t*
${\hat{a}}_{t}$	Human activity determined at time step *t* by recognition algorithm
*C*	Classification algorithm (binary classifier)
$d_{i,t}$	Binary decision related to data transmission from sensor node *i* at time step *t*
*D*	Time series of of binary decisions
*i*	Identifier of sensor node
*I*	Set of identifiers of sensor nodes
$i^{*}$	Identifier of cluster head
*M*	Recognition model used by cluster head to recognize human activities
$M_{i}$	Classification model used by sensor node *i* for selecting the data that have to be transmitted
*m*	Number of time steps
*n*	Total number of sensor nodes
*R*	Activity recognition algorithm
*S*	Multivariate time series of preprocessed sensor readings from *n* sensor nodes
$S^{\prime }$	Part of time series *S* used to train the recognition model *M*
S″	Part of time series *S* used to train the classification models $M_{i}$
$S_{t}$	Set of data transmitted to cluster head from sensor nodes at time step *t*
$S_{i,t}$	Set of preprocessed sensor readings collected by sensor node *i* at time step *t*
SM(Z)	Sub-model for recognition of human activity based on data from sensor nodes belonging to subset *Z*
*t*	Time step
*T*	Time period (ordered set of time steps)
